# Up Regulation of the Maternal Immune Response in the Placenta of Cattle Naturally Infected with *Neospora caninum*

**DOI:** 10.1371/journal.pone.0015799

**Published:** 2011-01-19

**Authors:** Anne Rosbottom, Helen Gibney, Peter Kaiser, Catherine Hartley, Robert F. Smith, Rebecca Robinson, Anja Kipar, Diana J. L. Williams

**Affiliations:** 1 Department of Veterinary Pathology, School of Veterinary Science, University of Liverpool, Liverpool, United Kingdom; 2 Institute for Animal Health, Compton, Berkshire, United Kingdom; 3 Department of Veterinary Clinical Science, Veterinary Teaching Hospital, Leahurst, Neston, United Kingdom; Louisiana State University, United States of America

## Abstract

*Neospora caninum* is an intracellular protozoan parasite which is a major cause of abortion in cattle worldwide. It forms persistent infections which recrudesce during pregnancy leading to foetal infection and in a proportion of cases, abortion. The mechanisms underlying abortion are not understood. In this study, recrudescence of a persistent infection in eight naturally infected cows occurred between 20 and 33 weeks of gestation. Animals were killed at the time of recrudescence and parasites were detected in the placentae and foetuses. An active maternal immune response consisting of an infiltration of CD4+ and CD8+ T cells and a 46–49 fold increase in interferon-γ and interleukin-4 mRNA was detected. Other cytokines, notably interleukin-12 p40, interleukin-10 and tumour necrosis factor-α were also significantly increased and Major Histocompatibility Class II antigen was expressed on maternal and foetal epithelial and stromal fibroblastoid cells. Significantly, despite the presence of an active maternal immune response in the placenta, all the foetuses were alive at the time of maternal euthanasia. There was evidence of parasites within foetal tissues; their distribution was restricted to the central nervous system and skeletal muscle and their presence was associated with tissue necrosis and a non-suppurative inflammatory response involving lymphocytes and macrophages, irrespective of the gestational age of the foetus. Whilst an active maternal immune response to a pathogen in the placenta is generally considered to be damaging to the foetal trophoblast, our findings suggest that the presence of a parasite-induced maternal immune response in the placenta is not detrimental to foetal survival but may contribute to the control of placental parasitosis.

## Introduction

Mammalian pregnancy has long been regarded as a form of ‘allogeneic graft’ with the foetus ‘parasitising’ the female uterus necessitating a suppression or modulation of the maternal immune response [1], [2]. More recently it has been suggested that the interaction between the foetal trophoblast and the maternal immune system is a supportive, regulatory interaction orchestrated by the foetal trophoblast with maternal immune cells such as uterine NK cells, dendritic cells and monocytes/macrophages recruited into the placenta to enable its development and function [3]. However in the presence of an infectious agent this relationship becomes perturbed, changing the phenotype of maternal immune cells from being tolerant into an aggressive or pro-inflammatory phenotype leading to foetal expulsion [4]. For example infection with pathogens such as *Listeria monocytogenes, Chlamydophila abortus, Brucella* spp or *Toxoplasma gondii* can have serious consequences in pregnancy [5]–[8]. These pathogens are normally controlled by a type 1 immune response and it has been suggested that activated maternal T cells and associated T helper 1 (Th1) cytokines, recruited to control infection, have a detrimental effect on foetal survival and/or placental function [9], [10].

*Neospora caninum* is an intracellular protozoan parasite, closely related to *T. gondii* and a leading cause of abortion in cattle world-wide [11]. Transplacental transmission (TPT) is a major feature of *N. caninum* infection and can occur following recrudescence of a persistent infection during pregnancy (endogenous TPT), or following *de novo* infection by ingestion of the oocyst stage of the parasite by a pregnant cow (exogenous TPT) [12]. Both forms of TPT can result in foetal death or birth of clinically normal but persistently infected (PI) calves. It is widely accepted that recrudescence of a persistent infection during pregnancy is the most common route of TPT and that the majority of persistently infected cattle acquire infection before birth [13].

The immune response to *N. caninum* is typically pro-inflammatory in nature involving interferon-γ (IFN-γ) and cytotoxic CD4+ T cells [14]–[17]. It has been suggested that a *N. caninum*-specific pro-inflammatory response in the placenta could lead to placental damage and foetal death through the effects of cytotoxic T cells and IFN-γ, TNF-α and IL-2 mediated destruction of foetal trophoblast cells [14]–[19].

It is well established that the time during gestation when foetal infection occurs is critical in determining the fate of the foetus [14]. Experimental infection of pregnant cattle with *N. caninum* in the first trimester of pregnancy (day 70/284) resulted in extensive foetal infection, tissue necrosis and death, but more immunologically mature foetuses survived a similar infection in late gestation (day 210/284) and show histological evidence of an immune response [20]. There were clear differences in the severity of pathological changes in the placenta, parasite loads and expression of both pro-inflammatory and anti-inflammatory cytokines depending on the time of infection; all were much greater following infection in the first trimester of pregnancy when foetuses died [21]. Therefore, interplay between both maternal and foetal immune systems has been suggested, with uncontrolled parasite replication within the immature foetus resulting in circulation of high numbers of parasites back into the placenta, which in turn leads to an enhanced maternal immune response [20], [21]. Nonetheless it is still unclear if it is the parasites themselves or the strong inflammatory immune response to the parasites which cause placental epithelial cell necrosis and ultimately leads to death of the foetus.

To address this question we have investigated the immune response, pathological changes and parasite loads in the placenta of naturally, persistently infected cattle at the time when parasite recrudescence and foetal infection occurred. Our findings show that in naturally infected cattle whose foetuses survive infection, significant maternal immune responses consisting of invading CD4+ and CD8+ T cells, expression of Major Histocompatibility Complex (MHC) Class II antigens and the presence of both pro and anti-inflammatory cytokines are detected in the placenta. The significance of our findings is that a significant anti-infectious immune response in the placenta is not necessarily detrimental to foetal survival. This has important implications for our understanding of immune events in the mammalian placenta and our interpretation of the role of the maternal immune response in pre-term births and abortion.

## Results

### Recrudescence of a persistent *N. caninum* infection was detected in eight pregnant animals but no foetal death occurred

**Figure 1:**
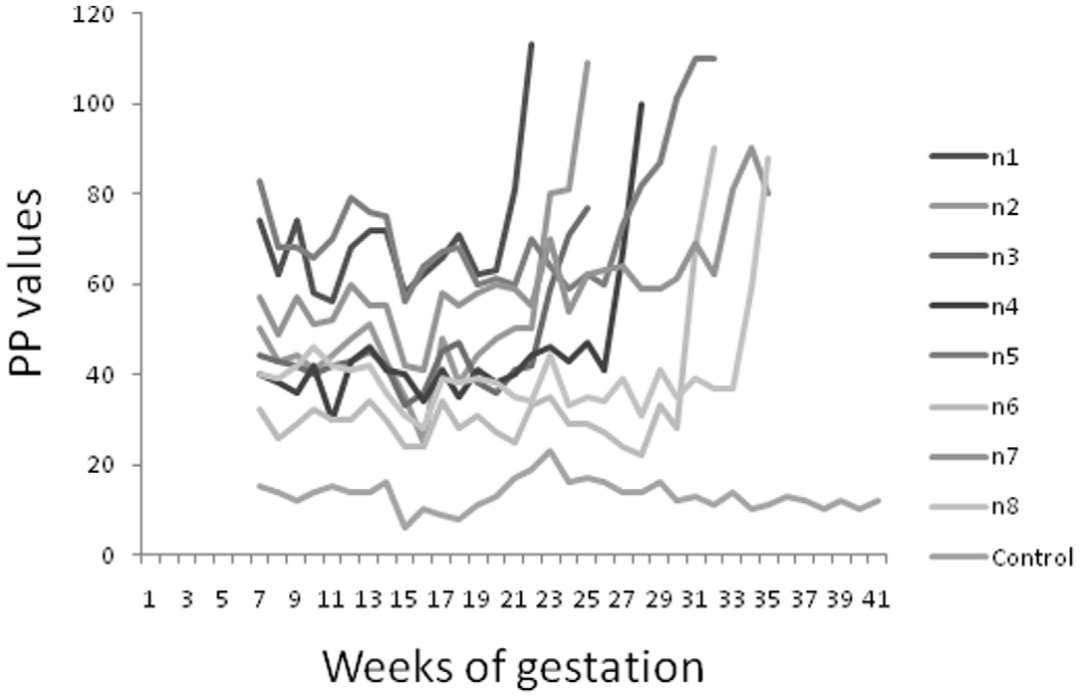
Antibody responses in persistently infected cattle during pregnancy. *N. caninum*-specific antibody responses were measured weekly in eight naturally, persistently infected cattle (n1–n8) during gestation. Results are expressed as percent positivity (PP) values which are calculated as a ratio of test optical density to the optical density of a high positive control [36]. A cut off value of 25PP was used to indicate an infected cow. All eight cows had positive values (>25PP) prior to artificial insemination. Antibody values were measured weekly as soon as pregnancy was confirmed at 6 weeks gestation. For each individual animal, antibody levels were considered to be rising when the PP value increased above the mean +95% confidence intervals calculated for the PP values for the first six serum samples taken (6–12 weeks gestation). Each cow was killed when the PP value increased to >50% higher than the mean (6–12 weeks) PP value. This ranged from two to five weeks from the time when an increase in PP value was first detected. The control represents the PP values for one uninfected sentinel cow, housed with the infected group.

Eight persistently infected cows each had positive serum antibody percent positivity (PP) values at 6 weeks of gestation with a median (range) of 47 (28–83). *N. caninum*-specific antibody levels increased sharply between week 20 and 33 of gestation (Figure 1, Table 1). Each of these animals was sacrificed between two and five weeks after the increase in antibody was first detected. Ultrasound scanning was used on alternate days and immediately before euthanasia to detect a foetal heartbeat and establish viability of each foetus. In all eight cows, the foetuses were alive immediately before euthanasia. The median (range) PP value was 90 (60–113) at euthanasia. Two of the eight cows were pregnant with twins thus 10 foetuses were examined.

**Table 1:** Evidence that *N. caninum* infection was present in the foetuses and placentae of all eight cows following parasite recrudescence.

Animal number	Week of gestation when evidence of recrudescence was first detected (week of euthanasia)	Detection of parasite DNA in foetal brain	Detection of parasites by immunohistology in foetus or placenta		Placental necrosis
			Foetus	Placenta	
1	20 (22)	+, +*	−	+ (6/10)	+
2	22 (25)	+	+	−	+
3	22 (25)	+	−	−	+
4	26 (28)	+	+	+ (1/10)	+
5	27 (32)	+	+	−	+
6	30 (32)	+	−	+ (1/10)	+
7	32 (35)	+, −*	−	−	+
8	33 (35)	−	+	−	+

Detection of parasites or parasite-associated necrosis in the placenta and foetal tissues of ten foetuses obtained from eight naturally persistently *N. caninum*-infected cattle killed two to five weeks after first detection of evidence indicating parasite recrudescence had occurred. All ten foetuses were alive when the dams were euthanized as shown by ultrasound scanning to detect foetal heartbeat. Tissues were obtained within one hour of death. For histology and immunohistology, 10 full-thickness pieces of placentome from each animal were collected and brain, heart, spinal column and liver were collected from the foetuses. Two cows were carrying twins thus ten foetuses were examined. No parasite DNA was detected by PCR in any placental tissue samples analysed.

*where two symbols are given this denotes a value for each twin.

Twelve non-infected, pregnant control cows were euthanized at 13 (n = 6) and 31 (n = 6) weeks of gestation respectively and their tissues used as uninfected controls. The foetuses of these control cattle were alive immediately before the dams were sacrificed.

### Evidence of *N. caninum* infection was detected in the foetuses and placentae in all eight cows following parasite recrudescence

**Figure 2:**
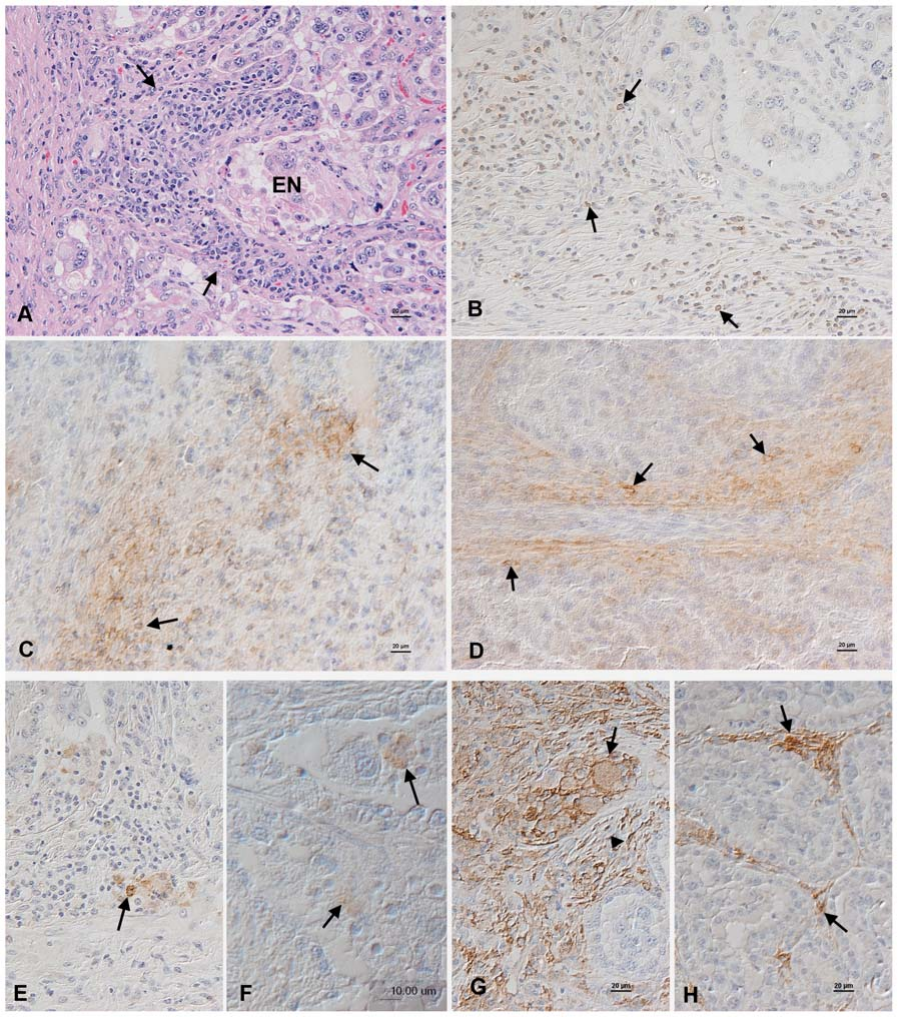
Histology and immunohistology in the placenta after parasite recrudescence. Placenta after parasite recrudescence. A. Animal no. 5, 32 weeks of gestation. There is a focal area of epithelial cell necrosis (EN) and a focal lymphocyte-dominated mononuclear interstitial inflammatory infiltrate (arrows). Haematoxylin-eosin stain. B–D. Animal no. 2, 25 weeks of gestation. The inflammatory infiltrate is dominated by CD3-positive T cells (B, arrows) which are comprised of CD4+ (C) and CD8+ (D) T cells (arrows). E. Animal no. 7, 35 weeks of gestation. Macrophages, as highlighted by their expression of myeloid/histiocyte antigen (arrow) are very rare in the inflammatory infiltrates. F. Animal no. 2, 25 weeks of gestation. *Neospora* antigen is observed within foetal chorionic epithelial cells (top arrow) and in cell free tachyzoites (bottom arrow) in areas of epithelial cell necrosis. G. Animal no. 2, 25 weeks of gestation. MHC Class II antigen expression is seen in foetal and maternal epithelial cells (arrow) and in stromal fibroblastoid cells (arrowhead). H. Control animal, 30 weeks of gestation. There is patchy MHC Class II antigen expression by stromal fibroblastoid cells (arrows). B–H. Peroxidase anti-peroxidase method, Papanicolaou's haematoxylin counterstain.

*N. caninum* DNA was detected in the brain and/or heart of eight foetuses (including one set of twins) and parasite antigen was detected by immunohistology in 4/10 foetuses and 3/10 placentae. In all eight cows there was evidence of parasites in either the placenta or foetuses and focal epithelial necrosis was evident in all ten placentomes examined for each animal (Table 1; Figure 2A, F). These results demonstrate that the parasite had invaded the placenta and crossed into the foetuses in all eight animals.

### Evidence of a maternal immune response was evident in the placenta of cattle following recrudescence of infection

**Figure 3:**
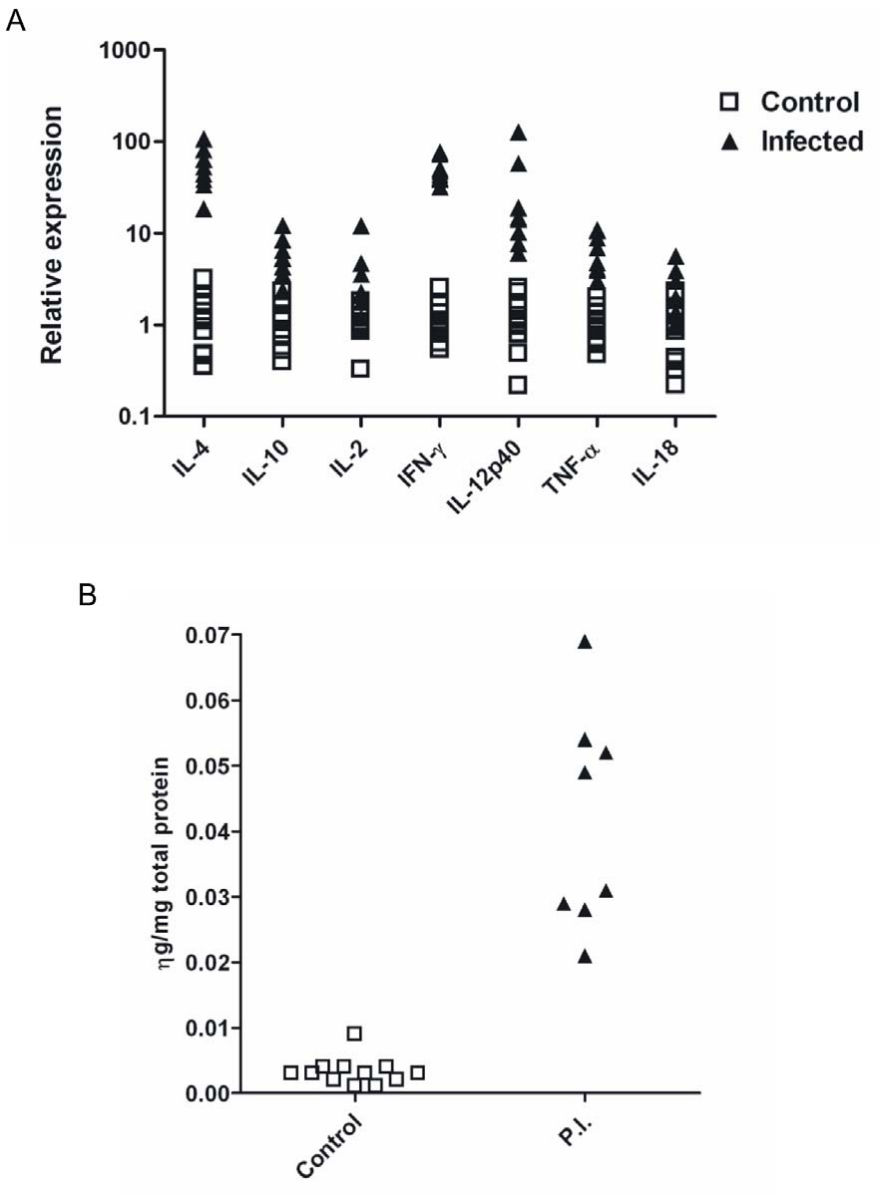
Cytokine responses in placenta of cattle following parasite recrudescence. A. Cytokine mRNA was measured in the caruncle tissue of eight naturally, persistently infected cattle two to four weeks after parasite recrudescence and placental infection. All cytokine mRNAs were significantly up regulated in caruncle from infected cows compared to 12 uninfected cows euthanized at 13 (n = 6) and 33 (n = 6) weeks of gestation. IL4 and IFN-γ were most highly up regulated (by 48.7 and 45.8 fold respectively p<0.0001). IL-12p40 increased by 14.6 fold, IL-10 by 5.4 fold and TNF-α by 5.9 fold (p<0.0001). IL-2 increased by 2.9 fold (p<0.01) and IL-18 increased by 1.9 fold (p<0.05). B. Interferon-γ protein levels extracted from placental tissue of eight cows following recrudescence of a naturally acquired, persistent *N. caninum* infection. IFN-γ was measured by ELISA using the Bovigam IFN-γ kit in fluid extracted from the caruncles of ten placentomes per animal. There was a highly significant increase in IFN-γ in the caruncles from infected animals – a median of 0.04ng/mg total protein extracted compared to 0.003ng/mg extracted from caruncles from uninfected cattle.

1. **Expression of IL-2, IL-4, IL-10, IFN-γ, TNF-α, IL-12p40 and IL-18 mRNA increased in the maternal placenta.** To determine if parasite recrudescence resulted in an immune response at the foetomaternal interface, we measured cytokine mRNA expression in the maternal placenta. Compared to 12 uninfected control animals, expression of all cytokine mRNA was significantly increased (Figure 3A). Most highly increased was IL-4 and IFN-γ mRNA by 48.7 (18.4–107.2) and 45.8 (32.2–76.5) fold respectively (P<0.0001). IL-12p40 increased 14.6 (6.1–127) fold; IL-10 and TNF-α increased by 5.4 (2.4–12.2) and 5.9 (3.1–10.9) fold, respectively (P<0.0001). Modest increases in IL-2 and IL-18 mRNA were detected; transcription of IL-2 increased by 2.9 (1.2–12.1) fold (P<0.01) and IL-18 increased by 1.9 (0.9–5.7) fold (P<0.05).
2. **An increase in placental IFN-γ protein was detected in persistently infected animals after parasite recrudescence.** IFN-γ and active TGF-β1 protein levels in the maternal placenta were measured by ELISA. There was a significant (P<0.001) increase in IFN-γ compared to the concentration in placental extracts from control animals (median 0.04 ng/mg total protein compared to 0.003 ng/mg in controls; Figure 3B). There was no significant difference between the levels of TGF-β_1_ in the maternal placentae of control and persistently infected animals.
3. **A mild to moderate lymphocyte-dominated cellular infiltrate consisting of CD4+ and CD8+ T-cells was detected in the placentae of infected cattle.** In all eight infected animals a mild to moderate multifocal lymphocyte-dominated, mononuclear interstitial infiltration was present; this was more pronounced in association with areas of necrosis of the maternal epithelium (Figure 2A). Infiltrating cells were predominantly CD3+ T-cells consisting mainly of CD4+ and slightly fewer CD8+ cells (Figure 2B, C and D). Staining for myeloid/histiocyte antigen identified very few macrophages among the infiltrating cells (Figure 2E) and B cells were seen very occasionally or not at all. In comparison control animals had very few scattered T cells (both CD4+ and CD8+) and occasional B cells and macrophages in the interstitium (data not shown).
  Placentomes of infected animals showed marked MHC Class II antigen expression by stromal fibroblastoid cells. Occasionally a focal area with positive maternal and/or foetal epithelial cells was seen as well (Figure 2G). In contrast only scattered stromal fibroblastoid cells expressed MHC Class II antigen in the uninfected controls (Figure 3H).

### Non-suppurative inflammatory lesions were present in 7/10 foetuses

**Figure 4:**
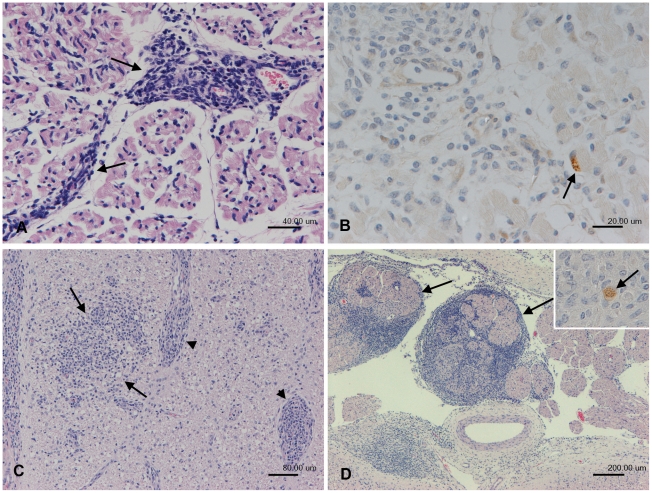
Histology and immunohistology of foetal tissues after parasite recrudescence. Foetus after parasite recrudescence. A, B. Foetus from animal no. 2, 25 weeks of gestation, skeletal muscle. A. Focal interstitial mononuclear (lymphocytes, plasma cells, macrophages) infiltration is seen (arrow; non-suppurative myosistis). B. *Neospora* antigen is seen within an intact myocyte immediately adjacent to the inflammatory infiltrate (arrow). C, D. Foetus from animal 6, 32 weeks of gestation. C. Spinal cord, exhibiting a moderate, focal extensive non-suppurative myelitis, represented by focal parenchymal (arrows) and perivascular (arrowheads) mononuclear inflammatory infiltrates. D. Dorsal root, spinal cord. Severe non-suppurative (mononuclear) radioculoneuritis (arrows). Inset: Staining for Neospora antigen identifies small extracellular cluster of tachyzoites within the inflammatory infiltrate (arrow). A, C, D. Haematoxylin-eosin stain. B, Inset D. Peroxidase anti-peroxidase method. Papanicolaou's haematoxylin counterstain.

Evidence that the foetus had mounted an immune response to the parasite was identified in 7/10 foetuses. The most commonly observed lesions were inflammatory changes in the skeletal muscles (7/7) and the nervous system (6/7). Mild to moderate focal or multifocal non-suppurative myositis (6/7), represented by interstitial infiltrates of macrophages, lymphocytes and plasma cells (Figure 4A) or a mixed cellular myositis with involvement of neutrophils (1/7) was seen. In one foetus, parasite antigen was detected within an intact myocyte immediately adjacent to the inflammatory infiltrate (Figure 4B).

Inflammatory changes in the nervous system included myelitis (6/6), encephalitis (4/6) and radiculoneuritis (3/6). In all but one foetus the inflammatory process was represented by a mild focal, partly perivascular, mononuclear infiltrate consisting of lymphocytes, macrophages and small numbers of plasma cells. The remaining foetus showed a more pronounced inflammatory reaction which also included a severe bilateral non-suppurative radiculoneuritis (Figure 4C & D). Parasite antigen was present in the spinal cord of one animal as two extracellular clusters of tachyzoites not associated with inflammatory infiltrates (Figure 4D inset). In addition two foetuses had a moderate multifocal non-suppurative myocarditis.

## Discussion

Here we show that in eight naturally, persistently infected cattle recrudescence of an existing *N. caninum* infection occurred between 20 and 33 weeks of pregnancy. There was evidence that the parasite had reached the placenta in all eight animals and was present in the tissues of nine out of ten foetuses at the time of euthanasia. Significantly all the foetuses were alive when the mothers were euthanized between two and five weeks after evidence of recrudescence was detected. In this experiment, recrudescence occurred after 20 weeks of gestation in all the animals; our previous work suggests that if recrudescence occurs before 20 weeks then foetal death ensues rapidly, but if recrudescence occurs in the second half of gestation, the foetus survives to term [22]. This is supported by experimental studies [14], [15], [23], [24] and so we have assumed that had the dams not been euthanized, the foetuses would have survived to term.

In the placenta of all eight cows there was evidence of limited parasite-associated necrosis in the foetal villi which was surrounded by an active maternal immune response. There was a mononuclear, T cell-dominated infiltration consisting of CD4+ and to a lesser extent CD8+ T cells and a highly significant increase in IFN-γ, both at the level of mRNA and protein expression. In uninfected cows, MHC Class II antigen expression was restricted to small numbers of fibroblastoid stromal cells whereas in infected animals MHC Class II expression was highly increased. In close proximity to areas of necrosis there were foci of MHC Class II-positive foetal and maternal epithelial cells. MHC Class II is not normally expressed on the foetal trophoblast [25]; in reports where MHC Class II expression has been detected in human placenta, this was associated with abortion [26]. In addition to expression of MHC Class II on foetal epithelial cells we also detected an highly significant increase in IFN-γ in the placenta following parasite recrudescence; IFN-γ induces MHC Class II expression on trophoblast cell lines [27] suggesting that the presence of IFN-γ in the placenta resulted in abnormal MHC Class II expression.

In addition to IFN-γ, other pro-inflammatory cytokines were also increased significantly, notably TNF-α, IL-12p40, with modest increases in IL-2 and IL-18. These results might suggest that a Type 1 pro-inflammatory response was elicited in the placenta in response to the presence of the parasite. However this was balanced by substantial and significant increases in IL-4 and IL-10, suggesting that the response in the placenta was not polarised towards either a Th1 or a Th2 phenotype. This supports previously reported results from experimental infections [21]. All these data suggest that there was an active maternal immune response in the placenta in response to the parasite including a 50-fold increase in IFN-γ but importantly this had not resulted in sufficient damage to the placenta to cause foetal death. The immunology of pregnancy is complex and our understanding of the interaction between the maternal immune system and the developing foetal trophoblast is still far from complete. IFN-γ is considered to be a necessary component of early pregnancy, limiting foetal trophoblast invasion, activating uterine NK cells, angiogenesis and inducing regulatory T cell differentiation [28], [29]. But there are numerous reports suggesting that the majority of abortions, preterm labour and foetal losses are associated with infections during pregnancy and the presence of maternal cytotoxic T cells and pro-inflammatory cytokines such as IFN-γ and TNF-α are a key part of the process leading to placental damage and foetal death [4], [30], [7]. Despite this wealth of information supporting the view that a pathogen-induced immune response in the placenta is detrimental to foetal survival, there are a small number of reports with conflicting evidence to suggest that the presence of pro-inflammatory cytokines in response to an infectious agent in the placenta may actually be beneficial [30]. For example Barber et al. [31] suggested that up regulation of IFN-γ was necessary and promoted foetal survival in mice infected with *Listeria monocytogenes*. IFN-γ is known to be important in controlling growth and spread of *N. caninum* [39] and detectable levels of IFN-γ in plasma of naturally infected cattle is associated with protection against abortion [40]. These observations support the role of IFN-γ in controlling *N. caninum* systemically. The data we present here suggest that an active maternal immune response to an intracellular infectious agent in the placenta although it was not polarised towards either a Th1 or a Th2 response, may have had an important role in controlling parasite growth.

Nine out of the ten foetuses in this study had been infected but parasites were restricted to the brain, spinal cord, dorsal root nerves, skeletal muscle and heart. Where parasites were detected or necrosis was observed, a non-suppurative inflammatory response was present indicating an active foetal immune response, even in the youngest foetus (22 weeks). Moreover there was no evidence of a correlation between the distribution and degree of inflammatory changes and the age of the foetus. Others have shown that the intensity of infection in aborted foetuses decreases with increasing foetal age, with significantly higher parasite loads in first trimester foetuses compared to second and third trimester foetuses [32]. Immunocompetence in cattle is thought to develop from about 100 days gestation and by 120 days gestation (17 weeks) the ability of foetal lymphocytes to respond to T cell mitogens and allo-antigens is well developed [33]–[35]. *Neospora* antigen-specific proliferation responses and IFN-γ and IL2 secretion are detectable in foetuses infected at 140 days (20 weeks) of gestation [24]. Previously we have shown that in very young foetuses (euthanized at 13 weeks gestation), parasites and parasite-associated necrosis are found widely disseminated throughout the foetus and most significantly are not normally associated with a foetal inflammatory response [20]. These data support the view that the foetal immune response could control parasite growth and drive differentiation of tachyzoites to bradyzoites, which ultimately results in persistent infection.

In conclusion, we have presented here a highly pertinent system that can be used to investigate events in the pregnant uterus in real time and with a representative biological system. Our results demonstrate that the presence of an active maternal immune response in the placenta is not in itself fatal to the foetus, although we cannot rule out the fact that the magnitude of the maternal response may be important. Nevertheless, these results taken as a whole suggest that foetal immune competence may also be a significant factor in determining whether or not abortion occurs.

## Materials and Methods

### Ethics statement

All animals used in this study were handled in strict accordance with good animal practice and the conditions defined by the United Kingdom Home Office under the Animals (Scientific Procedures) Act, 1986. The work was approved by the Home Office and carried out under Home Office Licence PPL40/2363 and PPL40/3005. All efforts were made to minimize suffering.

### Animals

Ten Friesian-Holstein cows were purchased from local farms that had a history of *Neospora*-associated abortion. All cattle had previously had a *N. caninum*-associated abortion and had positive *N. caninum* antibody values when tested using the Mastazyme *Neospora*-specific serum antibody ELISA (Mast Diagnostics, Liverpool, UK). The mean (95% C.I.) of the percent positivity (PP) value for 10 animals was 56 (41–70). A diagnostic value of ≥20PP is considered positive [36]. Cattle were negative for Bovine Viral Diarrhoea (BVD) antigen and vaccinated against *Salmonella* Dublin and Typhimurium (Bovivac S, Intervet, Milton Keynes, UK), *Leptospira hardjo* (Leptavoid-H, Schering-Plough Animal Health, Uxbridge, UK), BVD (Bovidec, Novartis Animal Health, Roysten, UK) and Infectious Bovine Rhinotracheitis (IBR) (Bovilis IBR marker, Intervet). Following testing and vaccination, cattle were housed in dog-proof accommodation. Oestrus was synchronised using progesterone-releasing intravaginal devices (PRIDs, CEVA Animal Health, Chesham, UK) and cattle artificially inseminated using standard techniques and were then kept with a bull for two weeks. Pregnancy was confirmed by transrectal ultrasonography at approximately day 35 of gestation.

### Detection of parasite recrudescence

A sudden, sharp increase in antibody levels was used as a measure of parasite recrudescence [22]. Heparinised blood samples were collected every week from 6 weeks of gestation and *N. caninum*-specific antibody values measured using the Mastazyme *Neospora*-specific serum antibody ELISA. Foetal viability was also monitored every week by detecting foetal heart beat by transrectal or transabdominal ultrasonography. Ultrasound monitoring of foetal heart beat was increased to 3 times weekly when levels of *Neospora*-specific antibody increased sharply. Cattle were euthanized for sample collection two to four weeks after the rise in *Neospora-*specific antibody was first detected.

### Tissue collection

Euthanasia was carried out using a captive bolt pistol followed by pithing; uterine and foetal tissues were collected within one hour of death. For analysis of cytokine expression by real-time reverse transcriptase-polymerase chain reaction (RT-PCR), full-thickness pieces of manually separated caruncle (maternal placenta) were placed in 5× volume of RNA-*later* (Sigma, Poole, UK). For ELISA, the same tissues were stored at −70°C. Caruncles consisted of >90% maternal tissue with a small amount of foetal villus tissue remaining in the crypts. For histology and immunohistology, 10 full-thickness pieces of placentome from each animal were collected into 4% buffered paraformaldehyde pH 7.4 (PFA) or embedded in cryoembedding gel (OCT, Tissue-tek, Pelco International, Redding, USA) and snap-frozen in isopentane cooled in liquid nitrogen. Foetal brain and spinal cord, an apical section of the heart, the lung, the main lobe of the liver, the spleen, the left kidney and adrenal gland, the pancreas, a section of jejunum, the quadriceps skeletal muscle, the femoral nerve, the umbilical cord, the bone marrow (BM) from the femur, the thymus, and mesenteric lymph node (MLN) were also collected into 4% PFA for histology and immunohistological identification of parasites. For detection of *N. caninum* DNA, further samples of foetal brain, heart and liver were collected aseptically and stored at −20°C.

### RNA extraction, reverse transcription and real-time PCR analysis of cytokine expression

RNA extraction, reverse transcription and real-time PCR analysis of cytokine expression was as described [21], [37]. Briefly, 100µg of placental tissue were homogenised into TRI reagent (Sigma), RNA extracted according to the manufacturer's instructions and 1µg of RNA reverse transcribed using M-MLV RNase-H^−^ Point Mutant reverse transcriptase enzyme (Promega, Southampton, UK), and random primers. Primers and probes were designed to be intron-spanning with the exception of 28S [21], [37]. Real-time PCR was performed using a Corbett Rotorgene 3000 in a 12.5µl volume using a qPCR probe master mix (Eurogentec, Seraing, Belgium) and FAM-TAMRA labelled probes. Six-point 10-fold dilution standard curves were generated for 28S, IL-2, IL-4, IL-10, IFN-γ, TNF-α, IL-12p40 and IL-18 using plasmid DNA and included, in duplicate, on each run. Relative copy numbers for samples, run in duplicate, were deduced from the standard curves and relative expression was calculated by normalising cytokine relative expression to that of 28S. Placental cytokine expression in infected animals was compared to that in 12 uninfected, pregnant cattle, six killed at 90 days of gestation and six killed at 230 days of gestation. Data were normalised to the median of relative expression in uninfected, pregnant controls, so the median of expression in the twelve controls = 1. cDNA samples from control and infected cattle were prepared in parallel and analysed on the same real-time PCR run.

### PCR for detection of *N. caninum* DNA

From each foetus, a 25 mg sample of brain, heart and liver was tested by PCR for the presence of *N. caninum* DNA. DNA was extracted and purified with a DNeasy kit (Qiagen, Crawley, UK) and PCR reactions carried out using the nested PCR method of Uggla et al. (38). Reactions contained 2.5 IU of HotStar Taq (Qiagen) and a final concentration of 1.5mM MgCl_2_. *N. caninum* tachyzoite DNA and ultrapure water were used as positive and negative controls, respectively. PCR products were resolved by agarose gel electrophoresis, stained using SYBR safe® DNA gel stain (Invitrogen, Paisley, UK) and visualised under ultraviolet light.

### Measurement of TGF-β_1_ and IFN-γ by ELISA

Tissue concentrations of TGF-β_1_ and IFN-γ in frozen, separated pieces of caruncle were measured by ELISA [21]. Briefly, tissues were homogenised in CelLyticMT Cell Lysis Solution (Sigma), and the supernatants assayed using the TGF-β_1_ E_max_® Immunoassay System (Promega) or the Bovigam IFN-γ ELISA kit (CSL, Victoria, Australia).

### Light Microscopy

Samples of placentomes and foetal tissues fixed in PFA for 24–48 h were routinely embedded into paraffin wax. Sections (3–5 µm thick) were prepared and either stained with haematoxylin-eosin for evaluation of histological changes, or used for immunohistology.

### Immunohistology

T cell (CD3, CD4 and CD8), B cell (CD79a) and monocyte/macrophage (myeloid/histiocyte antigen) markers were detected by immunohistology in two placentomes from each animal [21]. Monomorphic bovine MHC Class II antigen was detected using mouse anti-human HLA-DR (clone TAL.1B, Dako Cytomation). *N. caninum* antigen was detected in placentomes and foetal brain, heart and liver using anti-*N. caninum* antisera raised in rabbits [22]. Staining for CD3, CD79a, myeloid/histiocyte antigen, MHC Class II and *N. caninum* antigen was performed on formalin-fixed and paraffin-embedded tissue sections in which endogenous peroxidise was blocked before antigen retrieval. Staining for CD4 and CD8 antigens was performed on acetone-fixed frozen sections in which endogenous peroxidase was inactivated before staining.

Sections were incubated overnight at 4°C with primary antibody and for 30 min at room temperature with secondary antibody. Binding of the secondary antibodies was detected by the Vectastain ABC kit (Vector Laboratories, Peterborough, UK) or using the PAP method, with 3,3′-diaminobenzidine tetrahydrochloride (DAB, Fluka, Buchs, Switzerland) substrate before counterstaining with Papanicolaou's haematoxylin and mounting. Consecutive sections from each tissue were used as negative controls where the primary antibodies were replaced by TBS. A bovine lymph node served as the positive control for each marker.

### Statistical analysis

Statistical analysis of differences in cytokine expression was carried out with the Mann-Whitney U test using GraphPad Prism version 4.01 for Windows, GraphPad Software, San Diego, California, U.S.A., www.graphpad.com. Data are expressed in the text as median (range).
